# Living Arrangements as Dynamic Contexts for Cognitive Aging: The Protective Role of Social Participation

**DOI:** 10.1093/geroni/igaf122.4155

**Published:** 2025-12-31

**Authors:** Hyunjoo Lee, Sojung Park, Eunsun Kwon, Takashi Amano, Minyoung Kwak

**Affiliations:** Daegu University, Kyungsansi, Kyongsang-bukto, Republic of Korea; Washington University in St. Louis, St. Louis, Missouri, United States; Fairleigh Dickinson University, Florham Park, New Jersey, United States; Rutgers University Newark, Newark, New Jersey, United States; Daegu University, Gyongsan, Kyongsang-bukto, Republic of Korea

## Abstract

Living arrangements are a central part of older adults’ daily lives, shaping opportunities for social interaction, support, and cognitive stimulation. Prior studies show that co-residence with a spouse is often linked to better cognitive outcomes, while living alone or with non-spouse others may carry greater risks. Yet most research has focused on cross-sectional differences. Less is known about how changes in living arrangements over time affect cognition and whether these effects vary across subgroups. Using longitudinal data and fixed-effects models, this study examines the association between shifts in living arrangements and cognitive functioning, and the moderating role of social participation. We also examine whether the protective role of social participation differs by gender and education. Data come from 12 waves (2010–2022) of the National Health and Aging Trends Study (NHATS) in the U.S., including 39,676 observations from 6,295 adults aged 65 and older. Fixed-effects models assessed within-person changes in living arrangements, with sociodemographic and health characteristics controlled. Results indicated that transitioning from living with a spouse to living alone was associated with a 0.556-point decline in cognitive scores (p < 0.01). Each unit increase in social participation reduced this disadvantage by 0.108 points (p < 0.01). Social participation was also independently associated with higher cognitive scores (β = 0.110, p < 0.001). Subgroup analyses revealed stronger protective effects among men and those with higher education. These findings suggest that living arrangements are dynamic contexts shaping cognitive aging. Social participation represents a modifiable pathway to resilience, underscoring the importance of fostering engagement opportunities for older adults navigating household transitions.

